# Reduced airway levels of fatty-acid binding protein 4 in COPD: relationship with airway infection and disease severity

**DOI:** 10.1186/s12931-020-1278-5

**Published:** 2020-01-13

**Authors:** Lídia Perea, Ana Rodrigo-Troyano, Elisabet Cantó, Marisol Domínguez-Álvarez, Jordi Giner, Ferran Sanchez-Reus, Judit Villar-García, Sara Quero, Marian García-Núñez, Alicia Marín, Eduard Monsó, Rosa Faner, Alvar Agustí, Silvia Vidal, Oriol Sibila

**Affiliations:** 1 Inflammatory Diseases, Institut de Recerca de l’Hospital de la Santa Creu i Sant Pau, Biomedical Research Institute Sant Pau (IIB Sant Pau), Barcelona, Spain; 2 Respiratory Department, Hospital de la Santa Creu i Sant Pau, Biomedical Research Institute Sant Pau (IIB Sant Pau), Autonomous University of Barcelona, Barcelona, Spain; 3 Pneumology Department, Hospital del Mar, Institut Hospital del Mar d’Investigacions Mèdiques (IMIM), Autonomous University of Barcelona, Barcelona, Spain; 4 Centro de Investigación en red de Enfermedades Respiratorias (CIBERES), Instituto de Salud Carlos III (ISC III), Barcelona, Spain; 5 Microbiology Department, Hospital de la Santa Creu i Sant Pau, Biomedical Research Institute Sant Pau (IIB Sant Pau), Autonomous University of Barcelona, Barcelona, Spain; 6 Department of Infectious Diseases, Hospital del Mar, Institut Hospital del Mar d’Investigacions Mèdiques (IMIM), Barcelona, Spain; 7 Department of Respiratory Medicine, Parc Taulí Hospital Universitari. Institut d’Investigació i Innovació Parc Taulí, I3PT, Sabadell, Spain; 8 Respiratory Department, Hospital Universitari Germans Trias i Pujol, Fundació Institut d’Investigació Germans Trias I Pujol, Badalona, Spain; 9 Institut Respiratori, Hospital Clinic, Institut de Recerca Biomèdica August Pi i Sunyer (IDIBAPS), University of Barcelona, Barcelona, Spain

**Keywords:** FABP4, Chronic obstructive pulmonary disease, Macrophages, Bronchoalveolar lavage fluid

## Abstract

**Background:**

For still unclear reasons, chronic airway infection often occurs in patients with Chronic Obstructive Pulmonary Disease (COPD), particularly in those with more severe airflow limitation. Fatty-acid binding protein 4 (FABP4) is an adipokine involved in the innate immune response against infection produced by alveolar macrophages (Mɸ). We hypothesized that airway levels of FABP4 may be altered in COPD patients with chronic airway infection.

**Methods:**

In this prospective and controlled study we: (1) compared airway FABP4 levels (ELISA) in induced sputum, bronchoalveolar lavage fluid (BALF) and plasma samples in 52 clinically stable COPD patients (65.2 ± 7.9 years, FEV_1_ 59 ± 16% predicted) and 29 healthy volunteers (55.0 ± 12.3 years, FEV_1_ 97 ± 16% predicted); (2) explored their relationship with the presence of bacterial airway infection, defined by the presence of potentially pathogenic bacteria (PPB) at ≥10^3^ colony-forming units/ml in BALF; (3) investigated their relationship with the quantity and proportion of Mɸ in BALF (flow cytometry); and, (4) studied their relationship with the severity of airflow limitation (FEV_1_), GOLD grade and level of symptoms (CAT questionnaire).

**Results:**

We found that: (1) airway levels of FABP4 (but not plasma ones) were reduced in COPD patients vs. controls [219.2 (96.0–319.6) vs. 273.4 (203.1–426.7) (pg/ml)/protein, *p* = 0.03 in BALF]; (2) COPD patients with airway infection had lower sputum FABP4 levels [0.73 (0.35–15.3) vs. 15.6 (2.0–29.4) ng/ml, *p* = 0.02]; (3) in COPD patients, the number and proportion of Mɸ were positively related with FABP4 levels in BALF; (4) BALF and sputum FABP4 levels were positively related with FEV_1_, negatively with the CAT score, and lowest in GOLD grade D patients.

**Conclusions:**

Airway FABP4 levels are reduced in COPD patients, especially in those with airway infection and more severe disease. The relationship observed between Mɸ and airway FABP4 levels supports a role for FABP4 in the pathogenesis of airway infection and disease severity in COPD.

## Introduction

Chronic Obstructive Pulmonary Disease (COPD) currently is the third leading cause of mortality worldwide [1]. Chronic airway bacterial infection often occurs in COPD patients, particularly in those with more severe airflow limitation [2, 3]. The presence of airway infection increases the economic impact of the disease and worsens clinical outcomes including increased mortality [4]. Several studies have demonstrated that innate immunity alterations favor airway infection in COPD [5, 6]. However, the underlying biological mechanisms leading to chronic airway infection in COPD have not been yet fully elucidated.

Alveolar macrophages (Mɸ) are a central component of the innate immune response against airway infection. Several alterations of Mɸ in marker expression and functions have been previously described in COPD [7, 8]. Among many other functions, Mɸ produce fatty acid-binding protein 4 (FABP4), also known as adipocyte A-FABP or aP2. FABP4 is a member of the FABP family of small-molecular weight intracellular lipid chaperones that functions as a secreted adipokine and plays a role in airway defense against infection [9, 10]. For instance, in an experimental model of *Pseudomonas aeruginosa* infection in mice, the presence of FABP4 protected against airway infection [11]. However, limited data on COPD and its relationship with disease severity and airway infections are available.

We hypothesized that airway FABP4 levels may be reduced in COPD patients, especially in those with the more severe disease and with potentially more dysfunctional Mɸ. Accordingly, this study sought to: (1) compare airway (and plasma) FABP4 levels in COPD and healthy volunteers and, (2) study their relationship with presence of airway infection, quantity and proportion of Mɸ and several clinical markers of disease severity, such as the severity of airflow limitation (FEV_1_), the GOLD classification and the level of symptoms (CAT questionnaire).

## Methods

### Study design and ethics

This was a prospective, multicenter, cross-sectional study that included clinically stable COPD patients and healthy volunteers with normal lung function, who served as controls. Participants were recruited from five university tertiary hospitals [Hospital de la Santa Creu i Sant Pau (Barcelona, Spain), Hospital del Mar (Barcelona, Spain), Hospital Universitari Parc Taulí (Sabadell, Spain), Hospital Germans Trias i Pujol (Barcelona, Spain) and Hospital Clínic (Barcelona Spain)]. The study protocol was approved by the local institutional review board (IIBSP-MIC-2015-57) and all subjects gave signed informed consent.

### Participants

The diagnosis of COPD was established according to the Global Initiative for Chronic Obstructive Lung Disease (GOLD) guidelines [12]. The inclusion criteria were: 8 weeks of clinical stability (defined by the absence of an exacerbation that required oral corticosteroids and/or antibiotic treatment), age between 40 and 75 years, and FEV_1_ between 20 and 70% predicted. All patients underwent a computerized tomography scan, and those with bronchiectasis, lung cancer, pneumonia, and/or interstitial lung diseases were excluded. Other exclusion criteria were patients with active malignant disease and/or any type of immunosuppression, drug addiction or alcohol abuse. As controls, we included adult volunteers without respiratory diseases and with normal spirometry recruited in these same centers [13].

### Clinical assessment

Demographic data, the number of exacerbations in the previous year, the time from last exacerbation, relevant comorbid conditions and current treatments were recorded at inclusion using standardized questionnaires. All patients underwent spirometry (Datospir-600; Sibelmed SA, Barcelona, Spain) following international recommendations. Reference values were those of Mediterranean populations [14].

### Samples collection and processing

Bronchoalveolar lavage fluid (BALF), induced sputum and plasma were obtained from all participants and were processed immediately. BALF samples were recovered using 150 ml saline lavage with the bronchoscope wedged in the right middle lobe. BALF samples were centrifuged at 800 *xg* for 10 min to obtain the cellular pellet and the supernatant. Induced sputum was collected just before the bronchoscopy as previously described [15]. Sputum samples were disaggregated using dithiothreitol (Oxoid Ltd., Hampshire, United Kingdom) for 15 min and were centrifuged at 600 *xg* for 6 min to obtain the supernatant. Proteases inhibitors (Calbiochem, San Diego, CA) were added to the supernatants during thawing. Plasma samples were obtained from blood collected in ethylenediamine tetra-acetic acid (EDTA) tube and centrifuged at 850 *xg* for 10 min. BALF and sputum supernatants and plasma were stored immediately at − 80 °C until analysis.

### Microbiological study

Samples were processed for qualitative and quantitative bacteriology, as previously described [16]. Airway infection was defined as the presence of potentially pathogenic bacteria (PPB) at ≥10^3^ colony-forming units/ml in BALF [17] in clinically stable patients.

### FABP4 measurement

FABP4 levels were measured by validated, commercially available ELISA kit (RayBiotech, Peachtree Corners, GA) according to the manufacturer’s instructions. The limit of kit detection was 38 pg/ml. The dilutions used were 1/7 for BALF supernatants, 1/5 for sputum supernatants and 1/75 for plasma. BALF FABP4 levels were adjusted to the total protein content quantified using Qubit fluorometer (Invitrogen, Carlsbad, CA).

### BALF flow cytometry

In COPD patients, cellular pellet obtained from BALF samples was lysed to avoid red blood cells contamination (RBC lysing solution; BioLegend, San Diego, CA). Cells were resuspended in one ml of PBS supplemented with 2% bovine serum albumin (BSA; Roche Diagnostics GmbH, Mannheim, Germany) to quantify the number of total cells in a MACSQuant cytometer (Miltenyi Biotec, Bergisch Gladbach, Germany). Cells were adjusted to 1x10^6^cells/ml and stained for 15 min at room temperature in dark with viability dye (Zombie NIR; BioLegend), CD45-FITC, CD14-APC (Immunotools GmbH, Friesoythe, Germany) and CD15-PE (Biolegend, San Diego, CA). Aggregated and non-viable cells were excluded from the analysis. Mɸ were gated according to CD45 positive population, CD15 negative, CD14 positive and high side scatter (SSC) parameter.

### Statistical analyses

Results of continuous variables are presented as mean and standard deviation (SD) or median and interquartile range [25th – 75th percentile IQR], according to the Kolmogorov-Smirnov test of normality distribution, whereas categorical variables are presented as frequencies. Groups were compared using Student t-test, ANOVA test or their corresponding non-parametrical test when required. Correlations were analyzed using Spearman’s Rho due to the variables did not present a normal distribution. A *p*-value < 0.05 was considered significant. Statistical analyses were performed using SPSS version 22 and Graph Pad Prism 7 software.

## Results

### Characteristics of participants

Fifty-two COPD patients and 29 controls were included. Table 1 shows their demographic and clinical characteristics. COPD patients were older (65.2 ± 7.9 vs. 55.0 ± 12.3, *p* = 0.0002) than controls but gender (75.9 vs. 65.4% males, *p* = 0.6) and BMI (26.3 ± 4.7 vs. 28.7 ± 9.7, *p* = 0.7) were similar in both groups. Airflow limitation in patients with COPD ranged from mild to severe (FEV_1_ of 59 ± 15% of predicted) whereas spirometry was normal in controls by design. Twenty-four patients (46%) were classified as GOLD grade C and D, and 21 patients (40%) were frequent exacerbators, defined as those patients who suffered from 2 or more exacerbations during the previous year to the inclusion.

**Table 1:** Demographics and clinical characteristics among controls and COPD patients

	Controls (*n* = 29)	COPD (*n* = 52)	*P* value
Age	55.0 ± 12.3	65.2 ± 7.9	0.0002
Male, *n* (%)	22 (75.9)	34 (65.4)	0.6
BMI (kg/m^2^)	28.7 ± 9.7	26.3 ± 4.7	0.7
Smoking status, *n* (%)			
Never	5 (17.2)	0 (0.0)	< 0.0001
Former	10 (34.5)	41 (78.8)	
Current	14 (48.3)	11 (21.1)	
Pack-years	26.0 ± 19.0	43.5 ± 19.2	0.002
FEV_1_ (% pred)	97 ± 16	59 ± 16	< 0.0001
FVC (% pred)	92 ± 15	85 ± 16	0.07
FEV_1_/FVC	0.79 ± 0.05	0.52 ± 0.14	< 0.0001

Data is presented as mean ± SD unless otherwise indicated

Twelve COPD patients (23%) had airway infection. Patients with airway infection were predominantly males (92%) and older than non-infected ones (Table 2). *Haemophilus influenzae* was the most common PPB isolated (*n* = 9, 75%), followed by *Streptococcus pneumoniae* (*n* = 2, 17%) and *Moraxella catharralis* (*n* = 1, 8%).

**Table 2:** Patient demographics, clinical characteristics and prior treatments among non-infected and infected patients

	Non-infected (*n* = 40)	Infected (*n* = 12)	*P* value
Age	63.7 ± 7.6	69.9 ± 6.9	0.02
Male, *n* (%)	23 (57.5)	11 (91.7)	0.04
Smoking status, *n* (%)			
Never	0 (0)	0 (0)	0.7
Former	31 (77.5)	10 (83.3)	
Current	9 (22.5)	2 (16.7)	
Pack-years	42.3 ± 20.0	47.8 ± 16.1	0.2
Comorbid conditions, *n* (%)			
Cardiovascular	13 (32.5)	6 (50)	0.3
Hypertension	15 (37.5)	6 (50)	0.4
Diabetes	0 (0)	0 (0)	1
Gastroesophageal reflux	14 (35)	6 (50)	0.3
Treatment, *n* (%)			
ICS	22 (55)	6 (50)	0.5
LABA	32 (80)	8 (66.7)	0.3
LAMA	32 (80)	11 (91.7)	0.3
FEV_1_ (% pred)	58 ± 169	61 ± 17	0.6
FVC (% pred)	87 ± 16	76 ± 17	0.04
BMI (kg/m^2^)	26.4 ± 4.8	26.0 ± 4.4	0.8
GOLD stage, *n* (%)			
A	13 (32.5)	6 (50)	0.4
B	8 (20)	1 (8.3)	
C	9 (22.5)	1 (8.3)	
D	10 (25)	4 (33.4)	
Prior exacerbations, *n* (%)			
0	16 (40)	4 (33.3)	0.9
1	8 (20)	3 (25)	
≥ 2	16 (40)	5 (41.7)	
Weeks from last exacerbation	26.2 ± 18.9	27.3 ± 19.1	0.6
CAT questionnaire	11.2 ± 6.5	10.5 ± 7.6	0.6

Data is presented as mean ± SD unless otherwise indicated

### Airway and systemic FABP4 levels in patients and controls

**Fig. 1:**
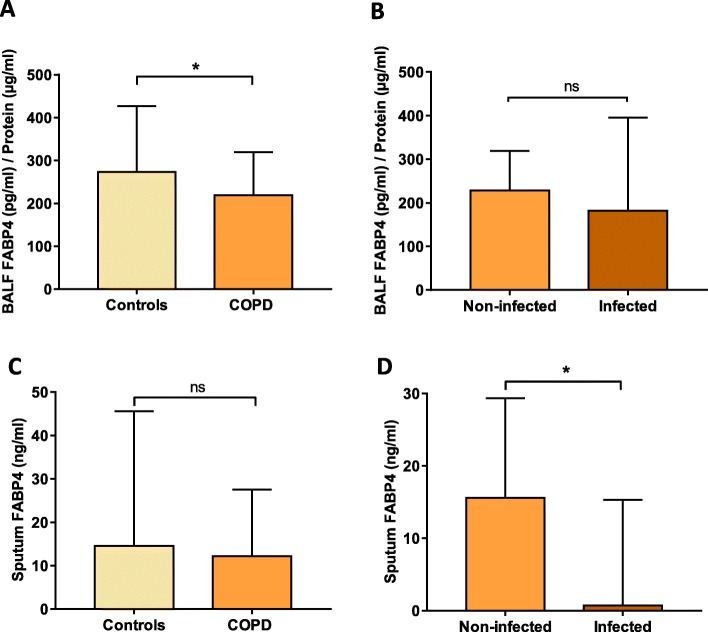
Airway FABP4 levels in COPD. **A** BALF FABP4 levels in controls and COPD patients and (**B**) in non-infected and infected patients. **C** Sputum FABP4 levels in controls and COPD patients and (**D**) in non-infected and infected patients. *P*-values were obtained by Mann-Whitney test. *p-value < 0.05. Data is represented as median with interquartile range

Airway FABP4 levels were lower in COPD patients than in controls, reaching statistical significant differences in BALF [219.2 (96.0–319.6) vs. 273.4 (203.1–426.7) (pg/ml)/protein, *p* = 0.03], but not in sputum [12.2 (0.7–27.5) vs. 14.5 (0.5–45.6) ng/ml, *p* = 0.4] (Fig. 1A, C). Yet, we observed a positive significant correlation between BALF and sputum FABP4 levels in the entire population (rho = 0.31, *p* = 0.01) and in COPD patients (rho = 0.37, *p* = 0.01). On the other hand, plasma FABP4 levels were similar in patients and controls [26.6 (19.3–39.5) vs. 25.2 (18.3–36.5) ng/ml, *p* = 0.4)] and no significant correlation was observed between systemic and airway FABP4 levels.

### FABP4 levels and airway infection in COPD patients

Airway FABP4 levels were lower in COPD patients with airway infection vs. those without it, reaching statistical significant differences in sputum [0.73 (0.35–15.3) vs. 15.6 (2.0–29.4) ng/ml, *p* = 0.02] but not in BALF [181.5 (28.7–395.5) vs. 228.5 (106.3–319.1) (pg/ml)/protein, *p* = 0.7) (Fig. 1B, D). No differences in plasma FABP4 levels were observed between COPD patients with and without airway infection [29.8 (20.4–45.0) vs. 26.1 (19.3–37.9) ng/ml, *p* = 0.6].

### FABP4 levels and disease severity

**Fig. 2:**
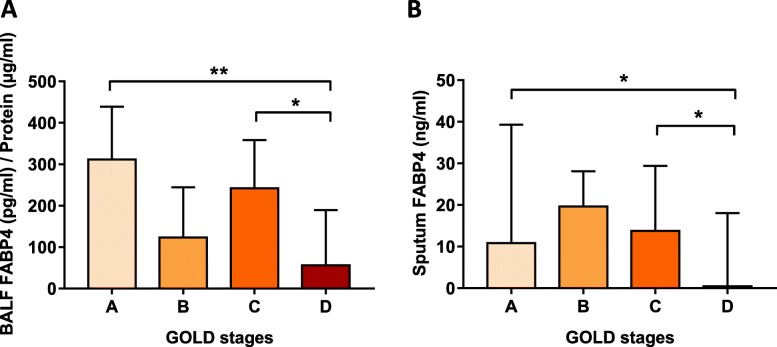
Association of airway FABP4 levels and disease severity. **A** BALF and (**B**) Sputum FABP4 levels and GOLD stages classified in mild (GOLD A), moderate (GOLD B), severe (GOLD C) and very severe (GOLD D). *P-*values were obtained by Mann-Whitney test. *p-value < 0.05 and **p-value < 0.01. Data is represented as median with interquartile range

**Fig. 3:**
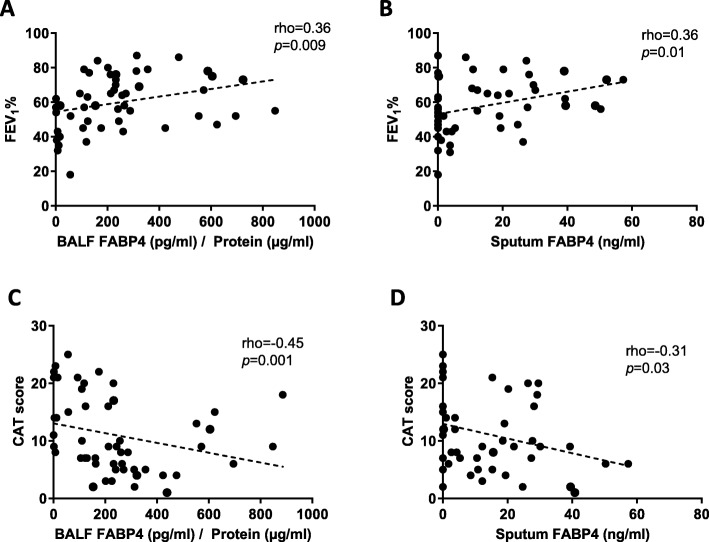
Relationship between FEV_1_ and (**A**) BALF FABP4 and (**B**) sputum FABP4. Relationship between CAT score and (**C**) BALF FABP4 and (**D**) sputum FABP4. Data was obtained using Spearman correlation

Airway (but not plasma) FABP4 levels were related with several measures of disease severity, including GOLD stage, airway limitation and quality of life. Patient with GOLD D had the lowest values both in BALF (Fig. 2A) and sputum (Fig. 2B). In addition, a positive correlation among airway FABP4 levels and FEV_1_ (% predicted) (Fig. 3A, B) and a negative correlation with levels of symptoms measured in CAT questionnaire (Fig. 3C, D) were observed.

No differences in airway FABP4 levels were observed regarding age and smoking status. Patients using inhaled corticosteroid (ICS) had lower values in BAL compared with those without ICS [(pg/ml)/protein, 123.6 (12.6–306.4) vs 250 (171.6–344.7), *p* = 0.03)].

### FABP4 and alveolar Mɸ

**Fig. 4:**
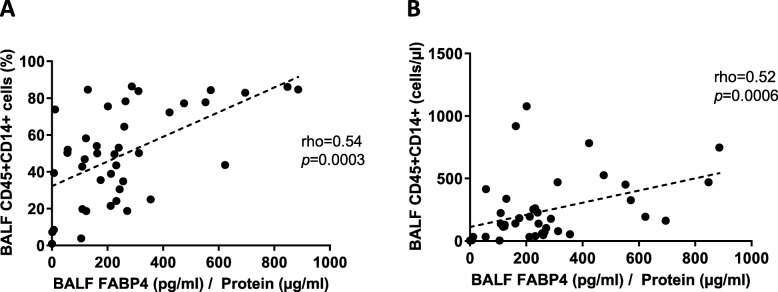
Relationship between BALF FABP4 levels and (**A**) proportion and (**B**) absolute number of BALF Mɸ. Data was obtained using Spearman correlation

Alveolar Mɸ represented 49.9 (26.4–76.8) % of cells in BALF of COPD patients, corresponding to an absolute number of 140 (48.9–311) Mɸ per μl. Both proportion and absolute number of alveolar Mɸ were significantly related with BALF FABP4 levels [proportion (rho = 0.54, *p* = 0.0003) and absolute number (rho = 0.52, *p* = 0.0006)] (Fig. 4). No significant differences among infected and non-infected patients and related to GOLD stage were found.

## Discussion

The main results of this study show that, as hypothesized: (1) airway (but not plasma) levels of FABP4 are reduced in COPD patients, particularly in those with airway infection and more severe disease; and, (2) BALF FABP4 levels are related to the number and proportion of alveolar Mɸ. Collectively, these results suggest that reduced production of FABP4 by alveolar Mɸ is associated with the presence of airway infection in COPD.

### Previous studies

FABP4 is an adipokine widely studied in metabolic and cardiovascular diseases [18]. Its role in chronic respiratory diseases is poorly understood, although it has been recognized that FABP4 is associated with airway inflammation [19]. Likewise, a dysregulation of airway FABP4 has been previously described in asthma [20] where it has been shown in experimental models that FABP4 participates in the recruitment and activation of eosinophils [21]. In patients with COPD, a dysregulation of systemic FABP4 compared with controls has been suggested [22], which is at variance with our findings here. Differences may be related to distinct inclusion criteria. Whereas Zhang et al excluded patients with metabolic and vascular comorbidities [22], known to be associated with elevated systemic FABP4 levels [23, 24], we did not because cardiovascular comorbidities are highly prevalent in patients with COPD (74% in our cohort) and we did not want to bias our study population. In any case, to our knowledge, our study is the first to investigate airway FABP4 levels, and their relationship with airway infection and disease severity in COPD.

### Interpretation of novel findings

Bacterial airway infections are relevant in the natural history of COPD [3] because it worsens clinical outcomes, including mortality and costs [4]. The molecular and cellular mechanisms that favor bacterial infection in some COPD patients are, however, not yet fully elucidated. We observed that airway FABP4 levels were reduced in COPD patients, particularly in those with evidence of airway infection, and we found a relationship between BALF Mɸ (absolute number and proportion) and FABP4 levels in COPD. Given that alveolar Mɸ produce FABP4 [9], these observations support a pathogenic role for a locally defective Mɸ production of FABP4. In support of this interpretation is the fact that FABP4 facilitates the interaction between Mɸ and neutrophils through the regulation of CXCL1, a chemokine secreted by Mɸ to recruit neutrophils to the site of infection [11, 25]. Further works studying Mɸ subpopulations would help to better understand the immunological mechanism related to FABP4 production by Mɸ.

On the other hand, we found that patients with severe COPD showed the lowest airway FABP4 levels. We also found that there was a direct relationship between airway FABP4 concentration and FEV_1_ levels and an inverse relationship between the former and CAT score (i.e. worse health status). In keeping with these observations, some previous studies had demonstrated that patients with severe COPD have altered airway innate immunity [16, 26, 27] that leads to chronic airway inflammation and dysregulation of normal alveolar Mɸ function [28]. Yet, because our study is cross-sectional, we cannot infer what is the cause and what is the consequence, this is, what comes first.

### Potential limitations

Our study has some limitations that deserve comment. First, controls were younger than COPD patients, but we did not observe any relationship between airway FABP4 levels and the age of the participants. Second, induced sputum was collected just before the bronchoscopy so we cannot discard any interaction with BALF samples, although this procedure had been applied to all the participants. Third, we did not perform flow cytometry in BALF from controls, but it is well reported in the literature that COPD alveolar Mɸ differ from healthy controls in phenotype [29] and in the ability to phagocytose bacteria and apoptotic cells [30, 31]. Forth, we have not determined FABP4 in infected patients without COPD and it would be of great interest to better understand its role in the pathogenesis or airway infection. Finally, we did not obtain follow-up samples, so we cannot infer if any therapeutic intervention (e.g., treatment with low dose azithromycin) can restore airway FABP4 levels and what clinical consequences that may have.

## Conclusions

FABP4 airway levels (but not plasma ones) are reduced in COPD patients, especially in those with chronic airway infection and more severe disease, in relation to a reduced number of alveolar Mɸ. These observations may be relevant for a better understanding of the pathogenesis (and eventual prevention or treatment) of chronic airway infection in these patients.

## Data Availability

All data generated or analyzed during this study are included in this published article.

## Abbreviations

APC: Allophycocyanin
BALF: Bronchoalveolar lavage fluid
BSA: Bovine serum albumin
CAT: COPD assessment test
COPD: Chronic obstructive pulmonary disease
EDTA: Ethylenediamine tetraacetic acid
ELISA: Enzyme-linked immunosorbent assay
FABP4: Fatty-acid binding protein 4
FEV_1_: Forced expiratory volume in one second
FITC: Fluorescein isothiocyanate
GOLD: Global Initiative for chronic obstructive lung disease
IQR: Interquartile range
PE: Phycoerythrin
PPB: Potentially pathogenic bacteria
SD: Standard deviation
SSC: Side scatter
